# Effects of Interlaminar Failure on the Scratch Damage of Automotive Coatings: Cohesive Zone Modeling

**DOI:** 10.3390/polym15030737

**Published:** 2023-01-31

**Authors:** Minfei Huang, Hanming Yang, Chenqi Zou, Mengyan Zang, Shunhua Chen

**Affiliations:** 1 School of Mechanical & Automotive Engineering, South China University of Technology, Guangzhou 510641, China; 2 School of Marine Engineering and Technology, Sun Yat-sen University, Zhuhai 519082, China; 3 Southern Marine Science and Engineering Guangdong Laboratory (Zhuhai), Zhuhai 519082, China

**Keywords:** automotive coatings, multilayer structure, scratch behavior, interface delamination, cohesive zone model

## Abstract

Interlaminar failure caused by scratches is a common damage mode in automotive coatings and is considered the potential trigger for irreversible destruction, i.e., plowing. This work strives to numerically investigate the mechanisms responsible for the complex scratch behavior of an automotive coating system, considering the interfacial failure. A finite element model is developed by incorporating a large deformation cohesive zone model for scratch-induced debonding simulation, where the mass scaling technique is utilized to minimize computational burden while ensuring accuracy. The delamination phenomenon of the automotive coating is reproduced, and its effects on scratch damage behavior are analyzed. Accordingly, it is revealed that the interlaminar delamination would produce significant stress redistribution, which leads to brittle and ductile damage of the coating and consequently affects the formation of plowing. Eventually, parametric studies on the effects of interfacial properties are performed. They demonstrate that the shear strength and shear fracture energy dominate scratch-induced delamination.

## 1. Introduction

The pursuit of aesthetic appeal in the exterior of a car has been a long-standing demand of consumers. Of the vast majority, automotive coatings are composite polymer films with a multilayer structure of clearcoat, basecoat, primer, electro coat, etc., which mainly provide vehicles with brilliant color and a glossy appearance, ref. [1] and also play an important role in protecting the underside of metal car bodies [2,3,4,5,6]. However, as car bodies are inevitably scratched in everyday use, scratch resistance is essential in guaranteeing the proper functioning of automotive coatings. Therefore, unveiling the internal mechanism of scratch damage has become an important and urgent concern for the performance improvement of automotive coatings.

Recently, a variety of instrumented tests were carried out to investigate fracture scratches in automotive coatings [2,3]. Feng et al. [7] conducted the nano-, micro-, and macro-scratch tests to gain a fundamental understanding of the scratch resistance of automotive clearcoats. Although the ASTM/ISO standards [8,9] were originally applied to the evaluation of bulk polymer scratch resistance [10], in recent years, they have been widely adopted for multilayer polymeric systems, including automotive coatings [11,12,13]. Following the standards, Hainsworth et al. [14] investigated the scratch-induced damage pattern of an automotive coating system with the use of scanning electron microscopy. Moreover, the ASTM scratch test was applied in the quantitative evaluation of the scratch properties of modified clearcoats [15]. Through these instrumented tests, it was revealed that automotive coatings are subjected to various scratch damage under high indentation load, including coating plastic deformation, surface cracking [7], and interlaminar delamination [14,16]. Although experiment results elucidated that scratch failure in automotive coatings has a tight relationship with the mechanical properties and the interlaminar adhesion, the intrinsic mechanism is still far from being clarified.

Numerical calculation methods provide further insights into the mechanism of polymer scratch behavior beyond the experimental approaches. For instance, the effect of material properties and internal structure on the scratch behavior of bulk polymers was studied through finite element (FE) simulations [17,18,19]. However, studies on modeling the scratching behavior of automotive coatings are scarce. Felder et al. [20] developed a 3D scratching FE model based on a rheological model and investigated the effect of hardening modulus on the scratch resistance of automotive body coatings. In recent work, our team utilized the FE simulation to analyze the damage mechanisms of scratching an automotive coating [16] and proposed a rate-dependent constitutive model to characterize the coating materials. However, the numerical model did not consider interface or material failure, and thus lacked an in-depth understanding of scratch damage. In addition to automotive coating systems, multilayer polymeric coatings are widely used in food packaging and protective coatings [21,22,23,24]. Scratch simulations of these coatings have been developed recently, and they offer heuristic guidance for the research into automotive coatings.

Scratch-induced damage in automotive coatings involves failure at the basecoat/primer interface, as emphasized in [7,16]. Scratches involving delamination usually create irreversible damage in the coating system compared to surface fractures, which are relatively easy to repair [5]. Generally, the weak interface bonding performance of multilayer polymeric structures makes them more vulnerable to scratch-induced delamination [25]. Several in-depth studies noted that the scratch behavior of multilayer polymer systems depends on the interfacial properties to some extent [26,27]. Some researchers focused on the trigger factors of interlaminar failure by analyzing the interfacial stress distribution at the onset of interfacial damage, without modeling the actual scratch-induced delamination process [28,29,30].

By way of contrast, the cohesive zone model (CZM) is a powerful tool to simulate the gradual scratch-induced debonding process [31,32]. Shu et al. [33] studied the interfacial damage behavior of hard diamond-like carbon (DLC)/steel systems in sliding contacts using cohesive zone elements (CZEs). Xu et al. [25] also simulated the delamination in alternating multilayer poly (methyl methacrylate) (PMMA)/polycarbonate (PC) materials during scratching based on CZEs. Nevertheless, the majority of research [31,32,33] on scratch-induced delamination has been concentrated on hard coatings. The common CZMs were implemented in the form of interface elements and were incapable of simulating the debonding process in soft coating interlayers due to the numerical problems in the condition of large deformation when using CZEs [34]. Some distinctive efforts were workable to avert these problems, including combining CZE with contact algorithms [35] and integrating cohesive constitutive relationships into the contact algorithm i.e., the contact/cohesive formulation [36]. However, as far as automotive coatings composed of softer materials are concerned, the performance of the former two methods in the delamination failure simulation is worth discussing.

This study aims at investigating interlaminar failure and its correlation to scratch damage in the automotive coating system through numerical simulation. The previous FE scratch model [16] is developed by incorporating a large deformation CZM, LDCZM [36], to simulate scratch-induced delamination. In order to reduce the high computing cost with the employment of explicit calculation, the mass scaling technique is applied to the simulation of polymer scratch behavior for the first time. The scratch-induced delamination process of the automotive coating system is well simulated. Furthermore, the effects of the interfacial properties on the delamination process have also been parametrically investigated at the end of the study. The main novelties of this paper can be summarized as follows:

- A finite element model for the scratch behavior of automotive coatings is developed, which accounts for the interlaminar failure of the coating system. This model is not only computationally efficient with the use of the mass scaling technique but is also verified to be effective by the good agreement between numerical and experimental results.
- The scratch-induced delamination phenomenon of an automotive coating is numerically reproduced, which makes it possible to understand its effects on coating scratch damage behavior via stress evolution analysis.
- The effects of the interfacial properties, i.e., interfacial strength and fracture energy on the delamination process have been numerically investigated.

The outline of the paper is as follows. Section 2 presents the numerical methods and the finite element model. Section 3 mainly describes the numerical investigation, including parameter determination, model validation, and a detailed analysis of the simulation results. The scratch test is also briefly introduced for better demonstration. Finally, a conclusion is drawn in Section 4.

## 2. Modeling Descriptions

### 2.1. Large Deformation Cohesive Zone Model

In this work, the numerical simulations were performed on an in-house explicit finite element code FEP-Fracture (A Finite Element Program for Fracture) based on Fortran 90/95 [37]. The emphasis of this work is to reproduce delamination that occurs during the scratching of automotive coatings via simulation. To this end, CZM is used at a potential failing interface to model the delamination process. As highlighted in the introduction, the most widely used CZMs have difficulties in characterizing failures of interfaces that are too soft and subjected to large deformation. In such cases, LDCZM [36], adopting the implementation of a contact-like algorithm, as suggested by Maleki-Jebeli et al. [38], is employed for the interface failure modeling of the automotive coating. In LDCZM, the traction forces are consistently calculated based on the mortar integration [39] of Gauss calculation points on slave segments, and thus a smoother cohesive-friction contact transition can be achieved. Furthermore, as validated in [36], the influence of interfacial element sizing on reaching a convergence resolution is less pronounced with the use of LDCZM, allowing for more freedom in mesh discretization.

The mixed-mode damage initiation is expressed in terms of a quadratic stress criterion, given by:

(1) $${\left(\frac{\langle T_{n}\rangle }{N}\right)}^{2}+{\left(\frac{T_{s}}{S}\right)}^{2}=1$$

where N and S are, respectively, the interfacial normal and tangential strengths, which are usually experimentally determined [40,41,42]; $T_{n}$ and $T_{s}$ are the normal and tangential traction component, respectively; and $\langle \cdot \rangle$ is the Macaulay bracket defined as $\langle x\rangle =\max(x,0)$. For the mixed-mode delamination, $\delta_{n}$ and $\delta_{t}$ are defined as the normal and tangential relative displacements on the interface, respectively. In particular, $\delta_{t}$ is incrementally updated based on the relative tangential velocities, allowing the accurate capture of historical displacement under large deformations.

**Figure 1:**
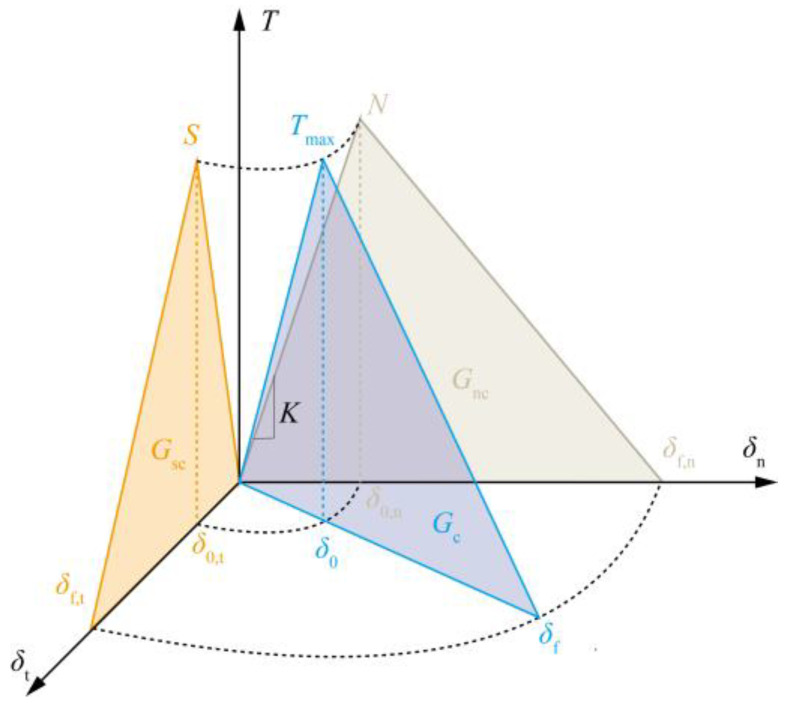
A typical bilinear mixed-mode traction-separation law for CZM.

A common bilinear mixed-mode traction-separation law (TSL) [43], as shown in Figure 1, is employed to characterize the cohesive constitutive relationship. The artificially introduced initial stiffness K ensures the load-bearing capacity of the interface before debonding. Generally, to avoid excessive penetration of the interface under compression, a relatively large K value is preferred. However, an overly large value would lead to the instability of the calculation, particularly in the case of soft material. With a reasonable value selected for the initial stiffness, the equivalent damage initiation relative displacement could be derived as:

(2) $$\delta_{0}=\left\{\begin{array}{cc} \delta_{0,n}\delta_{0,t}\sqrt{\frac{1+\beta^{2}}{\delta_{0,t}{}^{2}+\beta^{2}\delta_{0,n}{}^{2}}} & \delta_{n}>0\\ \delta_{0,t} & \delta_{n}\leq 0 \end{array}\right.$$

where $\delta_{0,n}=N/K$ and $\delta_{0,t}=S/K$ denote the equivalent damage initiation relative displacements in pure tensile and shear modes, separately, and $\beta =\langle \delta_{t}/\delta_{n}\rangle$ is a mixed-mode parameter.

When the equivalent displacement $\delta_{e}=\sqrt{{\langle \delta_{n}\rangle }^{2}+\delta_{t}^{2}}$ exceeds $\delta_{0}$, it indicates the onset of interfacial damage. For the prediction of delamination propagation, a famous power law is used [44], given by:

(3) $${\left(\frac{G_{n}}{G_{\mathrm{nc}}}\right)}^{\kappa }+{\left(\frac{G_{s}}{G_{\mathrm{sc}}}\right)}^{\kappa }=1$$

where $G_{\mathrm{sc}}$ and $G_{\mathrm{nc}}$ are the interfacial fracture energy per unit (critical energy release rate) in the tangential and normal directions, respectively, and κ is an empirical parameter. Then, the ultimate mixed-mode relative displacement can be deduced as [43]:

(4) $$\delta_{f}=\left\{\begin{array}{cc} \frac{2(1+\beta^{2})}{K\delta_{0}}{\left[\frac{1}{G_{\mathrm{nc}}^{\kappa }}+{\left(\frac{\beta^{2}}{G_{\mathrm{sc}}}\right)}^{\kappa }\right]}^{-\frac{1}{\kappa }} & \delta_{n}>0\\ \frac{2G_{\mathrm{sc}}}{S} & \delta_{n}\leq 0 \end{array}\right.$$

To assign the traction contribution, the damage variable is used to determine the interface damage evolution process and computed as:

(5) $$d=\left\{\begin{array}{cc} 0 & \delta_{e}<\delta_{0}\\ \frac{\delta_{f}(\delta_{e}-\delta_{0})}{\delta_{e}(\delta_{f}-\delta_{0})} & \delta_{0}\leq \delta_{e}<\delta_{f}\\ 1 & \delta_{f}\leq \delta_{e} \end{array}\right.$$

The effect of friction is particularly important in the simulation of interface failure in scratching since compression increases shear debonding resistance and friction plays a dominant role in this regard [45]. Thus, Coulomb’s friction law is incorporated into this model for friction characterization. To sum up, tangential and normal traction components regarding the friction effect are calculated as $T_{s}=(1-d)K\delta_{t}+dT_{\mathrm{friction}}$ and $T_{n}=(1-d)K\delta_{n}+dK\langle -\delta_{n}\rangle$, where $T_{\mathrm{friction}}$ denotes the friction stress calculated based on Coulomb’s friction law.

### 2.2. Mass Scaling

In this work, the mass scaling technique is utilized for the scratch simulation of polymer coatings in pursuit of lower time consumption. Usually, to perform FE simulations on the scratching of multilayer automotive coating systems, meshes with small thickness dimensions are needed due to the thin film structure, which also leads to a huge total number of elements. These result in a significant increase in the time necessary for explicit calculations. The basic concept of mass scaling is the adding of artificial mass via scaling up density to enlarge incremental time steps, thus reducing the overall computation time [46]. A particular formula is used to calculate a critical incremental time step that is established from the stability condition in an explicit scheme, given as:

(6) $$\Delta t_{\mathrm{cr}}=\frac{L}{c}$$

where L is the characteristic length of the smallest element in the current time; $c=\sqrt{E(1-\upsilon )/[(1+\upsilon )(1-2\upsilon )\rho ]}$ is the adiabatic sound speed; E is Young’s modulus; υ is Poisson’s ratio; and ρ is the material density. The given $\Delta t_{\mathrm{cr}}$ could be considered as the time required for the dilatational wave to travel through the smallest element. To maintain dynamic equilibrium at each time step, the incremental time step for the calculation should be less than or equal to $\Delta t_{\mathrm{cr}}$, which is set to 0.9 times the latter by default in the commercial FE package, LS-DYNA. By scaling up the density, the incremental time step is hence increased and overall computational time is accordingly reduced.

In the present study, the overall density of the coating material is scaled up to increase the critical time step. In general, mass scaling is feasible for situations where the physical response is less affected by inertia [47,48]. To verify the reliability of the application of this treatment in the simulation of coating scratches, the scaling factor is cautiously determined and the coating inertia effect on the scratch behavior is discussed later.

### 2.3. FE Scratch Model

In the scratch tests, 200 mm × 90 mm metal panel samples painted with commercial OEM automobile coating systems were employed. A spherical scratch tip with a diameter of 1 mm was used for the tests. Table 1 lists the basic information about the automotive coating system studied in this work, including the thickness and ingredients of each layer. The FE model was established in accordance with the coating system, and the loading and boundary conditions were the same as those of the experiment, which will be briefly introduced in Section 3.3. Scratch simulations on polymeric coatings have been carried out in numerous studies [16,21,49], and similar modeling techniques were utilized in this study.

**Figure 2:**
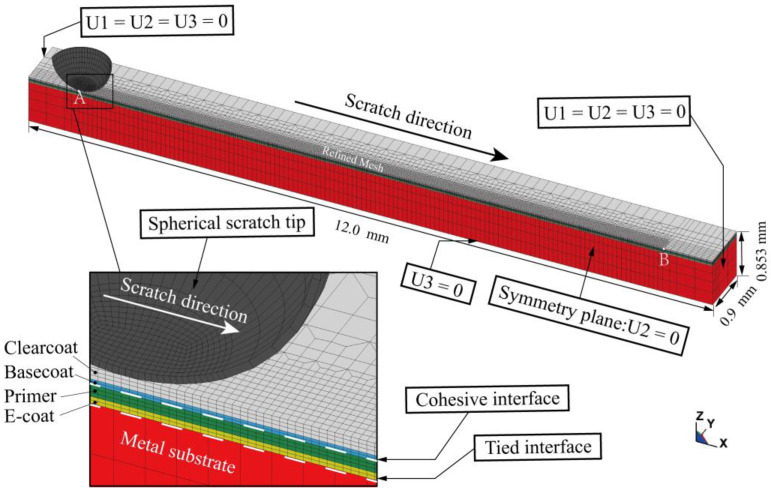
Scratch FE model of the automotive coating system, which could consider the interlaminar interface failure by the employment of the LDCZM.

Figure 2 demonstrates the meshes, dimensions, and boundaries of the FE calculation domain. A half model is established due to the symmetry along the scratch path. Nodes at the two ends were restricted in all degrees of freedom to simulate clamping conditions (U1 = U2 = U3 = 0), the bottom nodes were restrained in the *z*-direction to represent the supporting plane (U3 = 0), and nodes on the symmetric plane were restrained in the *y*-direction (U2 = 0). An element-based mortar contact algorithm [39] was utilized to consider the contact computation between the scratch tip and the coating surface, and the friction coefficient was set to 0.27 [50]. Owing to the adoption of a symmetrical model, the applied normal force was halved, linearly increasing from 0 to 15 N. The scratch speed was intentionally set as a constant value of 10 m/s to save computational time, and this treatment was the same as [51]. Eight-node linear brick elements were employed to describe the automotive coating system. To obtain the highest possible accuracy with guaranteed convergence, the number of elements for each layer in the thickness direction was 3, 2, 3, and 2, from top to bottom [16]. The refinement zone along the scratch path allowed a more precise capture coating deformation, with 512 elements along the length A–B [21]. It was proven that the substrate may not undergo significant deformation during the scratch process and was, thus, discretized with a coarse mesh to reduce the number of brick elements [52]. The tied interface algorithm handles the transition of meshes between the substrate and E-coat as a perfect bonding [16]. The employed LDCZM characterizes the interlaminar failure on the basecoat/primer interface. Within this work, the onset of delamination was considered at the moment that the first LDCZM calculation point on the cohesive interface failed, and the other interfaces between different layers were treated as perfect bonding. The introduction of a cohesive model at other interfaces was feasible, but not considered in this study for the following reasons. Firstly, experimental results suggested that interlaminar failure occurred at the basecoat/primer interface, and secondly, no possible stress incentives that may lead to delamination were observed at other interfaces where all interfaces were treated as perfect bonding in the previous simulation [16]. The established model eventually contained up to 72,724 elements and 94,077 nodes in total. In this work, the parameters for the interface failure were set to $G_{\mathrm{nc}}=G_{\mathrm{sc}}=100{\;J/m}^{2}$ and S=N=40 MPa [40]. The friction coefficient for the LDCZM interface was set as 0.25 [53].

A rate-dependent bilinear elastoplastic model [16] is used to characterize the mechanical response of polymeric coatings that are of great rate dependence, including clearcoat, basecoat, and primer. The material model considering the strain rate effect is expressed as:

(7) $$\left\{\begin{array}{l} \sigma_{0}(\dot{\varepsilon })=\sigma_{0}[1+k_{\sigma }\mathrm{lg}(\frac{\dot{\varepsilon }}{{\dot{\varepsilon }}_{\mathrm{ref}}})]\\ E_{0}(\dot{\varepsilon })=E_{0}[1+k_{E}\mathrm{lg}(\frac{\dot{\varepsilon }}{{\dot{\varepsilon }}_{\mathrm{ref}}})]\\ \sigma_{y}=\sigma_{0}(\dot{\varepsilon })+h\varepsilon_{p} \end{array}\right.$$

where $\sigma_{y}$ is the yield strength with hardening; $\varepsilon_{p}$ is the effective plastic strain; $\sigma_{0}(\cdot )$ and $E_{0}(\cdot )$ denote Young’s modulus and initial yield stress at the current strain rate $\dot{\varepsilon }$, respectively; $\sigma_{0}$ and $E_{0}$ are Young’s modulus and initial yield stress at the reference strain rate ${\dot{\varepsilon }}_{\mathrm{ref}}$, respectively; h is the hardening modulus; and $k_{\sigma }$ and $k_{E}$ are the rate-dependent coefficients.

The E-coat and the substrate, are modeled as perfect elastoplastic. The test data for calibration of the constitutive model were obtained from our former work [16]. In the uniaxial tensile tests for the clearcoat, the thin film specimens fractured without distinct hardening behavior. As a result, the hardening modulus of the clearcoat for this study was taken from [54]. The viscoelastic recovery and temperature effects were not considered in the present work. The steel scratch tip deformed negligibly in comparison to the coating and is thus modeled as a rigid body. All the material parameters are listed in Table 2.

## 3. Numerical Simulations and Discussion

### 3.1. Parameter Determination

As already stated, the cohesive interface stiffness K and the mass scaling factor will greatly affect the performance of the numerical model. Intending to minimize the calculation time while ensuring the calculation accuracy and convergence, these two parameters have to be duly selected.

To determine an appropriate K value, a set of simulations were employed. Table 3 lists the load for delamination onset with different K values. As a smaller K is selected, the CZM interface is softer and more susceptible to delamination. It is also found that when the initial stiffness is larger than 4.2 × 10^5^ MPa/mm, the calculation terminates soon after starting due to unreasonable oscillation occurring on the cohesive interface. However, it can be determined that the convergence results have already been obtained when the tested stiffness values ranged from 3.5 × 10^5^ MPa/mm to 4.2 × 10^5^ MPa/mm (the delamination loads differ less than 1.04%). Ultimately, 4 × 10^5^ MPa/mm was chosen as a satisfactory value for initial stiffness.

**Figure 3:**
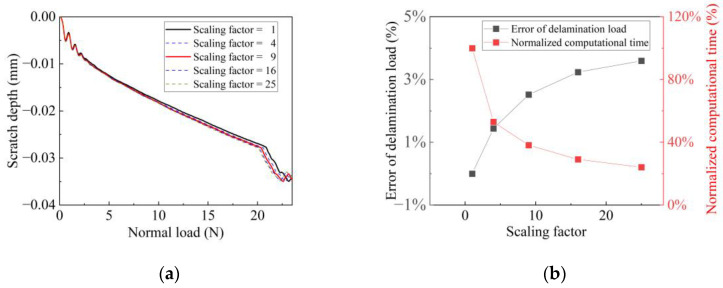
(**a**) Effect of mass scaling factors on scratch depth; (**b**) Errors in delamination load and reduction in computational cost with scaling factors.

In this study, the overall material density of the coating system was scaled up to increase the incremental time step, and thus save computational cost. To obtain an appropriate value, a set of simulations were carried out with the scaling factor values set as 1, 4, 9, 16, and 25. Figure 3a demonstrates the results by showing the simulated variation of scratch depth against loading normal force. The scratch depth curves exhibit sudden drops at a normal load of about 20 N. By comparing the simulation results, it can be confirmed that the inflection points of the curves also represent the onset of delamination. As seen from the figure, the scratch depth curves with different scaling factors exhibit a similar variation tendency, but differ slightly in the delamination load. The variation trend of the delamination load errors with the scaling factor is presented in Figure 3b. It is noticed that all of the FE calculations would be terminated due to excessive element distortion, which is a result of severe coating deformation after delamination. However, they stop at different moments as different scaling factors are employed. To obtain consistent and comparable results, the calculation times were recorded as the normal load reached 22.5 N. Without mass scaling, the total calculation time was 65.98 h using a desktop computer with an Intel(R) Core (TM) i5-10400 processor. For better demonstration and comparison, the reduction in computational time with scaling factors is also plotted in Figure 3b. The delamination load at the original density was 20.85 N. At scaling factors of 4, 9, 16, and 25, the computed critical loads of delamination were 20.55 N, 20.33 N, 20.18 N, and 20.10 N, which translate into percentage errors of 1.44%, 2.52%, 3.24%, and 3.60%, respectively. The calculation time, on the other hand, was accordingly reduced to 53.02%, 38.16%, 29.12%, and 24.10% of the unscaled time. It is, therefore, concluded that larger mass scaling factors would greatly reduce the calculation time while producing larger errors in simulation. However, the error is acceptable with a small scaling factor. Finally, a mass scaling factor of 9 was appropriately chosen for subsequent calculations on the premises of efficiency and accuracy.

### 3.2. Performance Comparison between LDCZM and CZE/Mortar Contact

**Figure 4:**
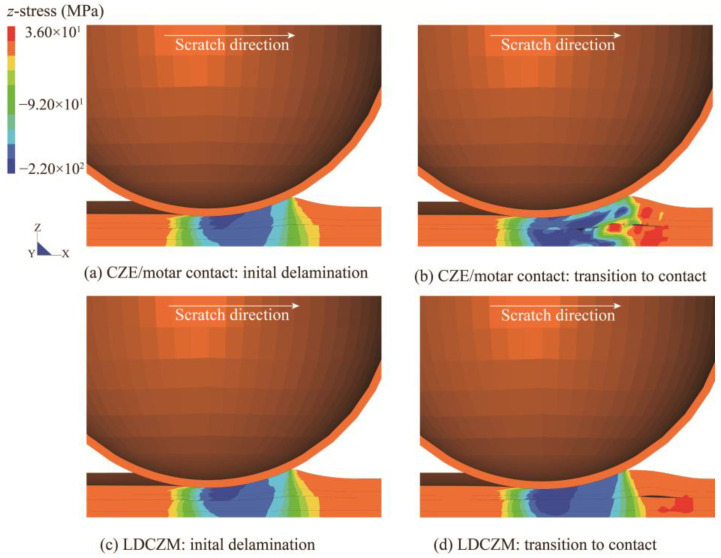
*z*-stress distribution predicted by (**a**,**b**) CZE/mortar contact and (**c**,**d**) LDCZM in scratch-induced delamination of the automotive coating system.

There are two distinct approaches to avert numerical problems that may occur when using common CZE for simulating large deformation delamination, as stated in the introduction. To intuitively illustrate the superiority of the employed LDCZM in simulating large deformation debonding between soft materials, a comparison between the performance of a CZM strategy [35] that combines CZE and mortar contact algorithm and an LDCZM in automotive coating scratch simulation was conducted. As the name implies, the CZE/mortar contact introduced a global contact treatment as the interface failure process. The difference between LDCZM and CZE/mortar contact lies in the way they compute cohesive traction force and the transition from cohesive to contact calculation. The *z*-stress distribution predicted with these two methods at a specific moment is shown in Figure 4. At the moment of initial delamination, these two methods produced virtually identical results, as illustrated in Figure 4a,c. Afterward, while LDCZM provides good interfacial stress compliance as delamination proceeds (see Figure 4d), the model using the CZE/mortar contact scheme oscillates at the interface (see Figure 4b). A possible key root of this problem may lie in the way the CZE scheme and mortar contact scheme compute traction force differs. Thus, a reasonably selected stiffness in the cohesive calculation may be inappropriate in the contact calculation, leading to a sudden change in traction force. The impact of this is amplified under the condition of a soft interface, and eventually forms a positive-feedback mechanism that causes oscillations.

### 3.3. Scratch Test and Model Validation

Before the simulation results are formally analyzed, the scratch test carried out to reproduce typical damage patterns on automotive coatings should be introduced. Scratch tests were performed according to the ASTM D7027/ISO 19252 standard [8,9]. A linearly rising normal load from 0 to 40 N was applied to the 200 mm × 90 mm metal panel samples through a spherical scratch tip with a diameter of 1 mm. The scratch speed was kept constant at 100 mm/s over a distance of 80 mm. Confocal microscopy was then used to record the damage patterns along the scratch path. It is worth noting that these were also the loading and boundary conditions that were referred to when building the simulation model. For a more detailed description, please refer to our previous work [16] in which scratch tests of automotive coatings were comprehensively carried out.

**Figure 5:**
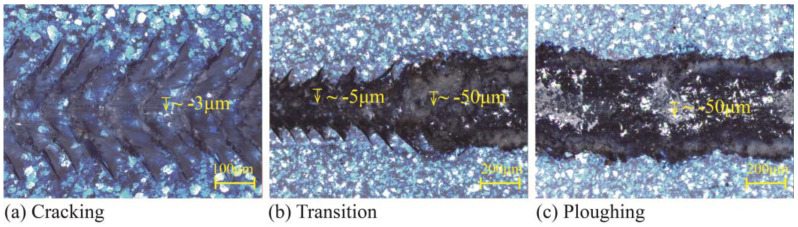
Typical scratch damage patterns of the automotive coating, (**a**) Cracking; (**b**) Transition; (**c**) Ploughing.

After tests, the typical damage patterns and surface topography along the scratch path were recorded, and are presented in Figure 5. The residual depths measured by confocal microscopy are marked. As previously summarized [16], the scratch process could be divided sequentially into three main stages based on the damage patterns, i.e., groove, crack, and plowing. At the initial groove stage, the scratch tip slides on the coating surface and results in a slight plastic deformation that is optically invisible. Later, the occurrence of the initial crack denotes the onset of the crack stage. The cracks gradually develop into a periodic and parabolic pattern, with the normal load continuing to increase, as shown in Figure 5a. It is determined that the critical normal load of cracking is around 10 N. The residual depth of the scratch decreases pretty slowly until the normal load reaches the plowing stage (see Figure 5b), a critical load of approximately 23 N. The residual depth at this stage exhibited a significant decline, and the upper layers of the coating were removed from the surface of the scratch path, which, consequently, exposed the primer underneath (see Figure 5c). At the plowing stage, the residual scratch depth is approximately 50 µm, which is close to the sum of clearcoat and basecoat thicknesses. It meant that interlaminar failure had occurred at the basecoat/primer interface, which is also the phenomenon that is of interest and simulated in this study. Similar damage patterns are also reported in other related research [7,15].

**Figure 6:**
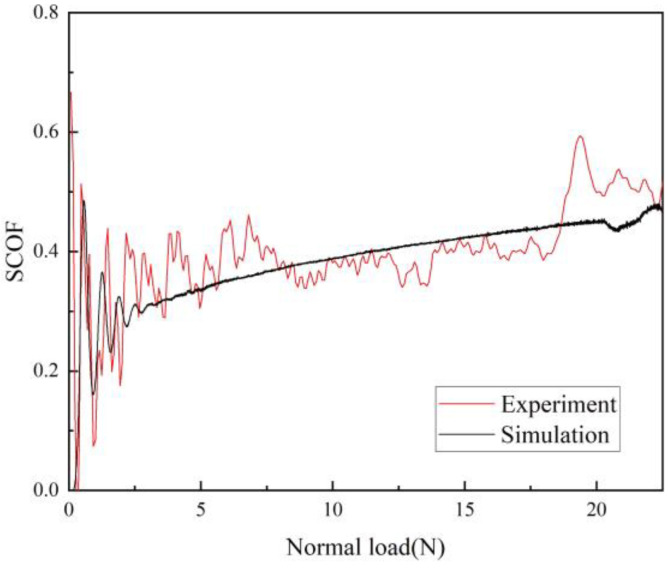
Scratch coefficient of friction (SCOF) as a function of normal load obtained experimentally and numerically.

To confirm the validity of the developed FE scratch model, the scratch friction coefficient (SCOF) obtained through the experiment was compared with the simulation outcome. The characterization of the scratch behavior of polymeric materials is challenging due to the high nonlinearity of their mechanical response [17]. The SCOF is influenced by the combination of surface friction and material pile-up during scratching, and it is thus capable of assessing whether a scratch model is quantitative and effective [51]. The simulated result compared well with the experimentally obtained SCOF data as illustrated in Figure 6. The fluctuations observed at the beginning of the curves are due to the inertia effect of the scratch tip movement. In this way, the reliability of the model is guaranteed.

### 3.4. FEM Findings

**Figure 7:**
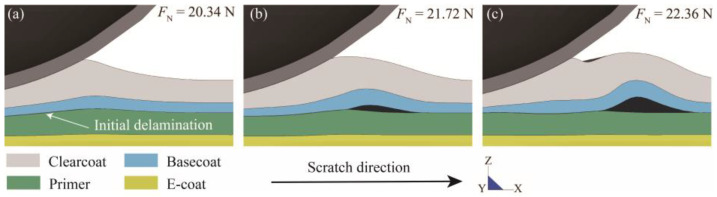
Longitudinal view of the delamination process at a normal load of (**a**) 20.34 N (Initial delamination), (**b**) 21.72 N and (**c**) 22.36 N.

Figure 7 intuitively presents the process of scratch-induced interlaminar delamination obtained via simulation. As the movement of the scratch tip proceeds, a notable tangential relative displacement is observed at the basecoat/primer interface. Once delamination occurs, the basecoat/primer interface remains unseparated due to the indentation from the tip (see Figure 7a), which suggests that the onset of interlaminar failure is caused by interface shear stress. Then, the delaminated area expands forward along the scratch direction, and coating buckling subsequently occurs ahead of the tip due to material flow (see Figure 7b,c). In addition, it is revealed that, as a result of coating buckling, the delamination process converts into mixed-mode debonding.

**Figure 8:**
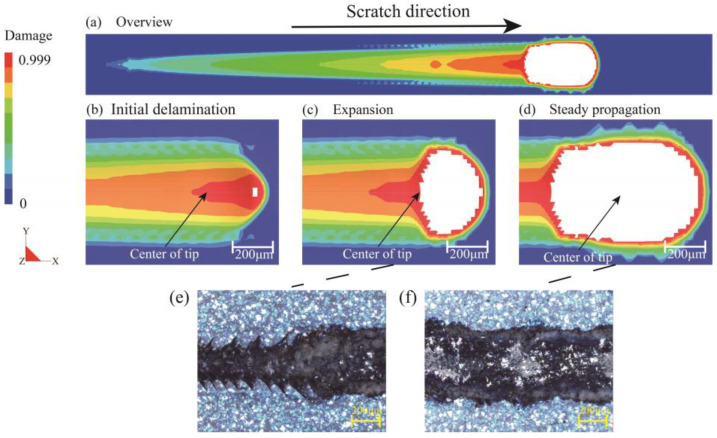
Top view demonstration of the delamination process. The damage variable evolution of the cohesive zone interface is presented. (**a**) Overview; (**b**) Initial delamination; (**c**) Expansion; (**d**) Steady propagation; (**e**,**f**) Experiment results.

Figure 8 exhibits the damage variable evolution of the LDCZM interface in the form of the interface element state variable, which distinctly lays out the process of delamination: from initiation to expansion, and then to steady propagation. When all cohesive calculation points on an interface element have failed, element deletion would be employed to represent the delaminated area. With the rising of normal load, the maximum value of the damage variables gradually increases until reaching 1 (see Figure 8b, which denotes the onset of delamination). Then, the width of the delaminated area rapidly increases (see Figure 8c) and finally maintains a certain value (see Figure 8d). At this time, the interface delamination process manifests a stable propagation state. The size of the simulated delaminated area is found to be in good agreement with the damaged region exposed in the plowing stage of the experimental result (see Figure 8e,f), which ulteriorly confirmed the reliability of the delamination process produced by simulation.

The above discussed the initiation and progress of the scratch-induced delamination process. However, no distinct correlation between interlaminar interface failure and material removal has been found through the above analysis. To more accurately represent the delamination and material removal process, the damage mechanism could be accounted for in the mechanical modeling of the polymeric coating. Although attempts were made in the scratch damage modeling of bulk polymers [17], it remains a challenging task in the case of multilayer polymeric coatings [25]. In the following, the stress evolution during delamination will receive attention to find the possible damage incentives, which would be of guiding significance for the in-depth simulation of scratch damage behavior in automotive coatings.

**Figure 9:**
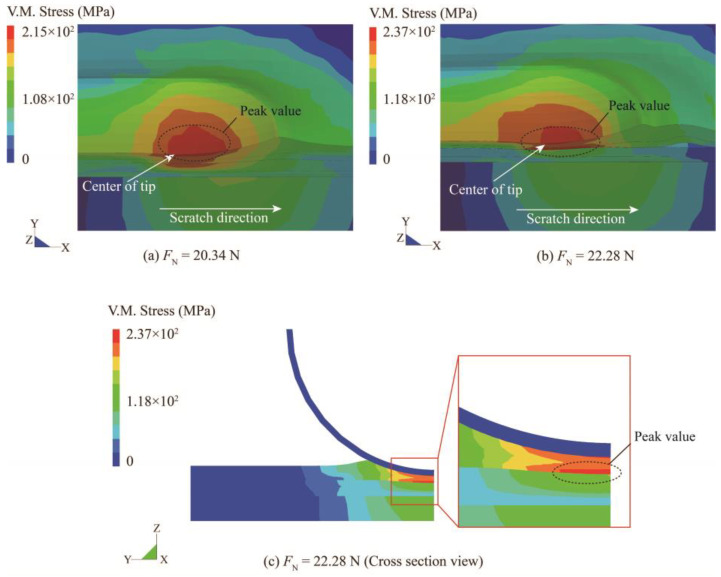
The von Mises stress contour beneath the scratch tip during scratching at the normal load of (**a**) delamination initiation; (**b**,**c**) plowing.

Figure 9 shows the von Mises stress contour at the normal load of 20.34 N and 22.28 N. For better visualization, the scratch tip in Figure 9a,b is hidden. Ductile failure is one of the typical damage types in amorphous polymers [17], and the von Mises stress could serve as an indicator of such a damage mode [55]. At a normal load of 20.34 N (when interlaminar failure initiates), the peak value of the von Mises stress is situated just below the scratch tip (see Figure 9a). After delamination initiation, the peak value of the von Mises stress is partially transmitted to the vicinity of the basecoat/primer interface, as shown in Figure 9b. To better illustrate this change, the model is cut along the center of the tip and displayed in a cross-sectional view (see Figure 9c). The high-stress concentration zone is also discovered in the basecoat in the cross-sectional view. It can be considered that this stress redistribution may lead to more severe ductile damage to the basecoat. This may explain the appearance of the cracks near the scratch center in Figure 5c. In addition, the delamination corresponds to a sudden drop in the scratch depth curves in Figure 3a, indicating the thinning of the upper layers. As a consequence, there is a higher possibility of the tip penetrating the upper layers and reaching the basecoat/primer interface after interlaminar interface failure.

**Figure 10:**
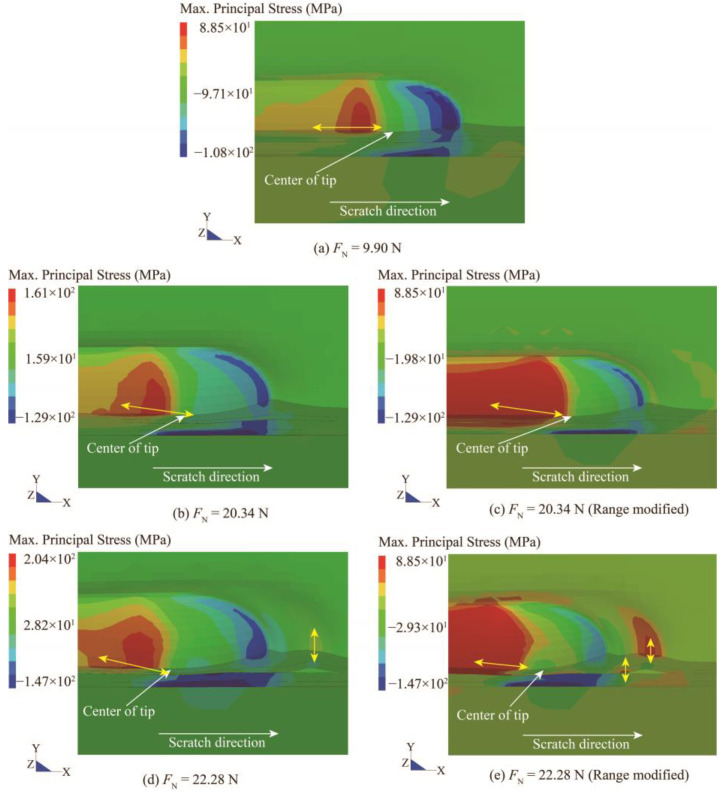
The maximum principal stress contour beneath the scratch tip during scratching at the normal load of (**a**) the onset of cracking; (**b**,**c**) delamination initiation; (**d**,**e**) plowing.

Figure 10 demonstrates the maximum principal stress contour at the normal load of 9.90 N, 20.34 N, and 22.28 N, respectively. Similar to the above, the tip is hidden, and the yellow arrows represent the direction of tensile maximum principal stress. The research in [56] suggested that the positive peak value of maximum principal stress is indicative of brittle failure (fracture) in amorphous polymers. Brittle damage may emerge in the position where the peak value of the tensile component of the maximum principal stress locates. In the experiment, it is obtained that the critical load for cracking is about 10 N. As presented in Figure 10a, there is a tensile stress concentration zone perpendicular to the scratch direction on the back of the scratch tip, which is considered to be the trigger for the onset of cracking. Similar conclusions were also given by Hossain et al. [21] and Zhang et al. [57] in their numerical studies on polymeric coating scratches. At the critical cracking load, the peak value of maximum principal stress is about 88.50 MPa. This value is thereby deemed sufficient to induce brittle damage in the coating and served as a damage initiation criterion. Then, the magnitude of the tensile stress gradually increases with the continued compression of the tip, resulting in periodic transverse cracks that gradually grow into a denser pattern (see Figure 5a). Figure 10b shows the contour of the maximum principal stress at the moment of initial debonding, which is similar to the contour at 10 N, except the peak value is larger. Afterward, as presented in Figure 10c, the alteration of the maximum principal stress arises along with the coating buckling, subsequently leading to delamination. To capture this alteration, the range of the legend value for the contour plot is set to a maximum limit of 88.50 MPa (see Figure 10b,d). With the above treatment, the deep red area indicates the position at which the coating is susceptible to fracture. As shown in Figure 10d, after the interface failure, the coating in front of the tip becomes an area prone to brittle failure. Therefore, this part of the coating may be taken away by the tip after damage, thus exposing the delamination area underneath.

Based on the FE results, the mechanism of scratch-induced delamination and post-delamination damage incentives of the automotive coating system were investigated. It was revealed that the delamination initiation was led by shear stress on the interface. Subsequently, the coating may be additionally damaged due to changes in the stress field induced by delamination and thus suffer material removal. The effect of delamination on the plowing formation could be summarized as follows: (1) severe ductile failure of the basecoat may facilitate the scratch tip to penetrate the thinned upper coating, and (2) the brittle damage of the coating that may occur in front of the tip would allow exposure of the delamination area beneath.

### 3.5. Parametric Study

**Figure 11:**
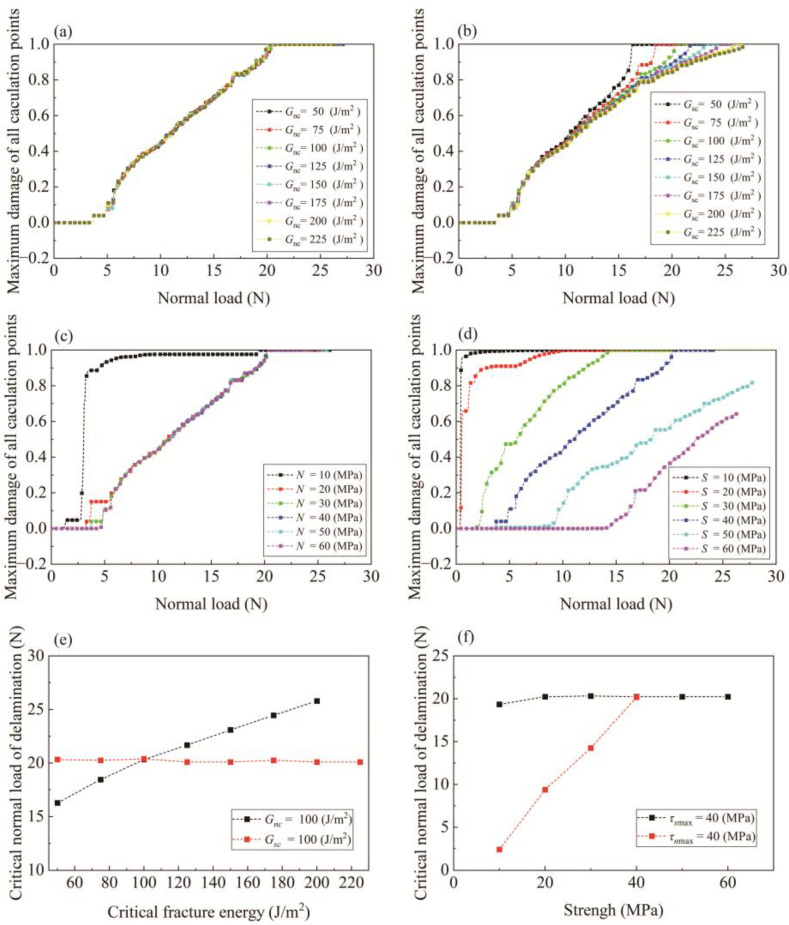
Effect of (**a**) $G_{\mathrm{sc}}$,(**b**) $G_{\mathrm{nc}}$, (**c**) *S* and (**d**) *N* on delamination process; Effect of (**e**) fracture energy and (**f**) strength on critical load of delamination.

Understanding the influence of interface properties on the delamination process is of great significance for improving scratch resistance. These properties include the interfacial strength and fracture energy in both the normal and tangential directions, which are parametrically investigated through a series of numerical simulations in this subsection. The basic concept is to study the influence of each parameter on the evolution of the maximum interface damage variable and delamination load. Specifically, the effect of strength is determined by varying the strength of one mode from 10 to 60 MPa in 10 MPa increments; consequently, the fracture energy was varied from 50 to 225 J/m^2^ in 25 J/m^2^ increments. The results are shown in Figure 11. The maximum damage in the figure relates to the maximum value of the damage variable of all of the calculation points on the cohesive interface. As mentioned in Section 2.3, when the maximum damage reaches 1, the onset of delamination is indicated.

It is revealed by Figure 11b,d–f that normal properties have little impact on the delamination process since the damage variable and critical normal load of delamination are substantially identical as *N* and $G_{\mathrm{nc}}$ increase. It must be noted that when the parameter was set to *N* = 10 MPa, the trend of the curve is quite different from the other curves, as shown in Figure 11d. This may be attributed to the employment of an overly low-strength value. In the implementation of LDCZM, the penalty method is adopted for the calculation of traction force [36]; however, this will generate spurious oscillation leading to unexpected damage to low-strength interfaces. Likewise, this phenomenon occurs at low shear strength (see Figure 11c). It is revealed in Figure 11a,c,e,f that shear properties significantly influence damage variable evolution and the delamination load. With increasing S and $G_{\mathrm{sc}}$, the capacity to resist delamination is enhanced. In particular, as the strength exceeds 40 MPa, no delamination is detected, even to the point when the calculation is terminated due to excessive element distortion.

Based on the above results, we may conclude that the scratch delamination of automotive coatings is shear-dominated, merely due to the enhancement of properties in the tangential direction contributing to the delamination resistance of the coating. In fact, this conclusion is closely relevant to the features of scratch deformation: indentation in the normal direction and tangential movement. Especially in thin coatings, the debonding interface is mainly compressed, making shear the only possible mode of delamination.

## 4. Conclusions

In this study, the interlaminar failure and its effect on the scratch damage of an automotive coating system were numerically investigated. Firstly, the scratch-induced delamination of the automotive coating system was effectively simulated using a CZM-based FE model. Then, the correlation between interlaminar failure and plowing damage of the automotive coating was analyzed via the observation of stress evolution. Finally, the effect of interface properties on delamination was investigated numerically. The main conclusions are given as follows:

1. With the aid of LDCZM, the developed FE model is capable of reproducing the delamination phenomenon when an automotive coating system is scratched. Although LDCZM enables the simulation of interfacial debonding between soft materials, nonphysical results appear at low interface strength. As a result, further development of the interface model is required to expand its application.
2. The severe scratch damage of the automotive coating system was closely related to the interlaminar failure. It was found that the delamination would induce significant stress redistribution resulting in brittle and ductile damage of the coating, and, consequently, affecting the formation of plowing.
3. In the automotive coating system, scratch-induced delamination was dominated by the shear strength and fracture energy. Therefore, enhancing these properties of the basecoat/primer interface could improve scratch resistance.

Ultimately, the model developed in this work does not simulate coating damage, hence actual material removal could not be reproduced in the simulation. Intralayer damage to coatings will be considered in future research.

## Figures and Tables

**Table 1:** The automotive coating system studied in the present work.

Layer	Thickness (μm)	Main Ingredient
Clearcoat	34	Solvent-based acrylic resin
Basecoat	15.2	Waterborne acrylic resin
Primer	33.4	Waterborne polyester resin
E-coat	19.9	Epoxy resin

**Table 2:** Material parameters of the automotive coating scratch model.

Part	Constitutive Model	Key Parameters
Scratch tip	Rigid body	E=20,000 MPa^1^, $\rho =1300\;\mathrm{kg}/m^{3}$,
Clearcoat	Rate-dependent bilinear elastoplastic	$E_{0}=768.95\;\mathrm{MPa}$, $\sigma_{0}=19.93\;\mathrm{MPa}$, ${\dot{\varepsilon }}_{\mathrm{ref}}=3.33\times 10^{-4}\;s^{-1}$,$k_{E}=0.26$, $k_{\sigma }=0.50$, ν=0.30, $\rho =1325\;\mathrm{kg}/m^{3}$, h=31.32 MPa
Basecoat	Rate-dependent bilinear elastoplastic	$E_{0}=466.84\;\mathrm{MPa}$, $\sigma_{0}=9.54\;\mathrm{MPa}$, ${\dot{\varepsilon }}_{\mathrm{ref}}=3.33\times 10^{-4}\;s^{-1}$,$k_{E}=0.34$, $k_{\sigma }=0.56$, ν=0.30, $\rho =903\;\mathrm{kg}/m^{3}$, h=60.48 MPa
Primer	Rate-dependent bilinear elastoplastic	$E_{0}=135.50\;\mathrm{MPa}$, $\sigma_{0}=2.434\;\mathrm{MPa}$, ${\dot{\varepsilon }}_{\mathrm{ref}}=3.33\times 10^{-4}\;s^{-1}$,$k_{E}=3.55$, $k_{\sigma }=3.46$, ν=0.35, $\rho =1633\;\mathrm{kg}/m^{3}$, h=91.40 MPa
E-coat	Perfect elastoplastic	E=2900 MPa, $\sigma_{y}=80.4\;\mathrm{MPa}$, ν=0.35, $\rho =1435\;\mathrm{kg}/m^{3}$
Substrate	Perfect elastoplastic	E=20,000 MPa, $\sigma_{y}=200\;\mathrm{MPa}$, ν=0.30, $\rho =7800\;\mathrm{kg}/m^{3}$

^1^ The elastic modulus is only used for determining the penalty stiffness for possible contact calculation.

**Table 3:** Influence of initial stiffness on the delamination load.

Initial Stiffness (1 × 10^5^ MPa/mm)	Normal Load (N)	Difference (against 20.25)
2.0	18.00	11.11%
2.5	18.75	7.41%
3.0	19.44	4.00%
3.5	20.04	1.04%
4.0	20.25	0
4.2	20.25	0
4.5	The calculation is incomplete.	

## Data Availability

Not applicable.
